# The Stem Sap Flow and Water Sources for *Tamarix ramosissima* in an Artificial Shelterbelt With a Deep Groundwater Table in Northwest China

**DOI:** 10.3389/fpls.2022.794084

**Published:** 2022-03-04

**Authors:** Feiyao Liu, Quangang You, Xian Xue, Fei Peng, Cuihua Huang, Shaoxiu Ma, Jing Pan, Yaofang Shi, Xiaojie Chen

**Affiliations:** ^1^Key Laboratory of Desert and Desertification, Northwest Institute of Eco-Environment and Resources, Chinese Academy of Sciences, Lanzhou, China; ^2^College of Resources and Environment, University of Chinese Academy of Sciences, Beijing, China; ^3^Drylands Salinization Research Station, Key Laboratory of Desert and Desertification, Northwest Institute of Eco-Environment and Resources, Chinese Academy of Sciences, Lanzhou, China; ^4^Arid Land Research Center, Tottori University, Tottori, Japan

**Keywords:** *Tamarix ramosissima*, stem sap flow, meteorological factors, water resources, arid region

## Abstract

The shelterbelt forest between oases and the desert plays a vital role in preventing aeolian disasters and desertification in arid regions of northwest China. *Tamarix ramosissima* (*T*. *ramosissima*), a typical perennial and native xerophyte shrub in Northwest China, grows naturally and is widely used in building artificial shelterbelt forests. The balance between water consumption and the availability of water determines the survival and growth of *T*. *ramosissima.* How *T. ramosissima* copes with extremely low rainfall and a deep groundwater table remains unknown. To answer this, the transpiration and the water sources of *T*. *ramosissima* were investigated by the heat balance and oxygen isotopic analysis method, respectively. Our results show that the daily *T*. *ramosissima* stem sap flow (SSF) was positively correlated with air temperature (Ta), photosynthetically active radiation (PAR), and the vapor pressure deficit (VPD). We found no significant relationship between the daily SSF and soil moisture in shallow (0–40 cm) and middle (40–160 cm) soil layers. Oxygen isotope results showed that *T. ramosissima* mainly sources (>90%) water from deep soil moisture (160–400 cm) and groundwater (910 cm). Diurnally, *T*. *ramosissima* SSF showed a hysteresis response to variations in PAR, Ta, and VPD, which suggests that transpiration suffers increasingly from water stress with increasing PAR, Ta, and VPD. Our results indicate that PAR, Ta, and VPD are the dominant factors that control *T. ramosissima* SSF, not precipitation and shallow soil moisture. Deep soil water and groundwater are the primary sources for *T*. *ramosissima* in this extremely water-limited environment. These results provide information that is essential for proper water resource management during vegetation restoration and ecological reafforestation in water-limited regions.

## Introduction

Aeolian disasters in arid regions are a major hazard to crop production and human life (Lei et al., 2003; Dong, 2004; Wang et al., 2011). Shelterbelt forests play an important role in weakening wind erosion, reducing sand sediment, and preventing aeolian desertification (Ryszkowski and Kedziora, 2007). In 1978, the Chinese government and some non-governmental organizations began one of the largest shelterbelt forest projects of the world. After four decades, the afforestation has reached 46.14 million hectares, increasing the local coverage from 12 to 22% (Wang F. et al., 2020). The large water deficit in arid regions and the urgency with which environmental protection is required make it challenging to allocate the scarce water resources for socioeconomic development without compromising the health of the ecosystem (Wang et al., 2017). Recent studies have demonstrated that soil water and groundwater have been strongly affected by the large-scale artificial shelterbelt forest creation, which has driven changes in evapotranspiration and infiltration (Wang et al., 2010; Jia and Shao, 2014; Jia et al., 2017) in the arid region and which may cause the further degradation of oasis shelterbelts (Fu et al., 2016).

Williams et al. (2004) illustrated that 95% of precipitation that reaches dryland ecosystems is lost through evapotranspiration and transpiration accounts for about 64% of total evapotranspiration globally (Good et al., 2015). Evapotranspiration is usually studied by using methods such as lysimeter measurements, the Bowen ratio, and eddy covariance approaches (Cassiani et al., 2015; Kucera et al., 2017; Jin et al., 2018; Nyabwisho et al., 2020). All of these methods can provide important information about ecosystem responses to environmental variability, but none of these approaches can be used to accurately evaluate the water consumption of individual plants. Accurate estimation of plant transpiration is helpful for understanding plant physiology and hydrology processes (Robertson et al., 2009; Hayat et al., 2020). The stem sap flow (SSF) is an essential indicator of water movement in plants and can be used to indicate plant transpiration (Yue et al., 2006). This method has been widely used in recent plant transpiration studies (Johnson et al., 2002; Lambs and Berthelot, 2002; Chirino et al., 2011; Steppe et al., 2015).

The main limitations for transpiration are the available energy and the evaporative demand of the ecosystem (Novick et al., 2016; Grossiord et al., 2017). An increase in available energy is expected to increase evaporative demand because of the higher air temperature (Ta), which affects transpiration. Increasing Ta and evaporative demand strengthens the driving forces for plant transpiration (Shen et al., 2015; Zha et al., 2017). Soil moisture is another major restricting factor for transpiration in moisture-stressed regions (Zhao and Liu, 2010; Gao et al., 2016). An increase in soil moisture can alleviate water stress and increase plant transpiration (Gao et al., 2016; Zha et al., 2017). A decrease in soil moisture can increase the hydraulic resistance between the soil and the root system, inhibiting water movement between the soil and the plant leaves, which can then trigger stomatal closure and so induce or enhance transpiration (Cochard et al., 2002). Studies into the effects of increased soil moisture on transpiration have reached inconsistent conclusions. For example, Zhao and Liu (2010) found that the SSF velocity increased significantly for *Nitraria sphaerocarpa* and *Elaeagnus angustifolia* following rainfall, due to the increased soil moisture. Several studies have documented that the SSF is driven by meteorological factors in arid areas and is only weakly affected by soil water, if at all (Bovard et al., 2005; Ma et al., 2017; Han et al., 2019). These seemingly conflicting findings suggest that the response of transpiration to soil moisture may depend on the availability of soil water and on the water use strategy of plant.

Precipitation is highly variable (Weltzin and Tissue, 2003; Zhao and Liu, 2010) and so moisture levels in the uppermost soil layers are extremely low and have strong variability in desert regions (Wang et al., 2017). Under such conditions, plants adopt conservative water use strategies and appear to exploit water from deep soil layers and groundwater (Xu and Li, 2006; Chen et al., 2017; Li et al., 2019; Pan Y.-X. et al., 2020). Modeling studies have suggested that groundwater uptake provides more than 80% of the total water intake used for transpiration (Gou and Miller, 2014; Yuan et al., 2014; Wang et al., 2018) and an increase in the groundwater table decreases plant transpiration rates (Nippert et al., 2010). Despite the recognized importance of groundwater in hyper-arid regions, most previous studies have focused on regions with a shallow (1–3 m) groundwater table (Gou and Miller, 2014; Wang and Pozdniakov, 2014; Wang et al., 2018) and few studies have focused on regions with groundwater depths of around 10 m. In addition, the regions where the groundwater table is shallow, soil water is recharged via capillary and hydraulic redistribution (David et al., 2013) and it is, therefore, difficult to discriminate the independent effects of groundwater on transpiration.

Stable isotope tracing is one of most effective tools for identifying the linkages between plant ecology and physical hydrology processes. Due to the fractionation, it does not occur, as stable isotopes migrate in plants, which have proved to be particularly effective for investigating the hydrological processes. Thus, the stable isotope characteristics of terrestrial ecosystem can provide distinct trace information for water exchange between soil, plant, and atmosphere in the terrestrial ecosystem (Pan Y.-X. et al., 2020), which has been extensively utilized to quantify plant water sources (Li et al., 2019; Dong et al., 2020; Pan Y.-X. et al., 2020).

*Tamarix ramosissima* (*T*. *ramosissima*) is a native desert shrub that is well adapted to drought and salinity stress and has been widely used in afforestation efforts in the transition zone between oasis and desert in the arid regions of northwest China (Xia et al., 2021). Precipitation increased in northwest China during 1960–2010 (Li et al., 2012); however, a large area of shelterbelt, which is dominated by *T*. *ramosissima*, has experience degradation since the 1960s (Zhang Y. et al., 2018). The groundwater table in some desert areas has fallen to 10–20 m due to industrial and agricultural expansion in the upper reaches of the river and the overexploitation of groundwater in nearby oases (Qu et al., 2007). The SSF of *T*. *ramosissima* was measured by Xu et al. (2008) and Li et al. (2015) and they found meteorological factors, not moisture in the uppermost soil layers, to be the primary drivers for the SSF in non-irrigated shelterbelt areas. These results implied that groundwater is the main water source for *T*. *ramosissima* in arid regions; however, there is no direct evidence to prove this hypothesis. To answer this, we quantify the transpiration and examine specific water sources for *T. ramosissima* by using the stable oxygen isotope methods in an area with a deep groundwater table (>9 m). We focus on the capacity of *T. ramosissima* for groundwater uptake, which has been neglected in previous studies.

In this study, meteorological factors, soil water, SSF, root distribution, and water sources for *T*. *ramosissima* were measured over the growing season in 2020. The goals of this study are to quantify the SSF for *T*. *ramosissima*, to analyze and identify environmental drivers for the SSF, and to identify the contributions of deep soil moisture and groundwater to *T*. *ramosissima* transpiration.

## Materials and Methods

### Study Site

**FIGURE 1:**
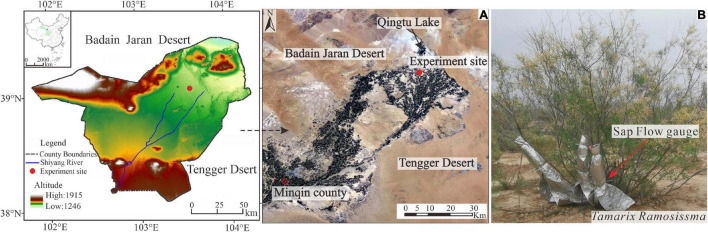
The location of the experiment site in the desert-oasis transition zone in Minqin, China **(A)** and the representative *Tamarix ramosissima* (*T*. *ramosissima*) shrubs and the equipment for the stem sap flow (SSF) measurements **(B)**. Modified from Pan J. et al. (2020).

This study was conducted in a downstream region of the Shiyang River (Figure 1), which is the third largest inland river in China. The study site is surrounded by the Badain Jaran and Tengger deserts and experiences intensive wind and sand activity. This region has a typical continental desert climate. The mean Ta was 8.9°C during 1981–2010, ranging from –8.1 in January to 23.6°C in July. The mean annual precipitation (P) is around 113.2 mm and precipitation is concentrated in June to September. The mean annual potential evapotranspiration (ET_0_) is 1383.0 mm and the resulting dryness index (ET_0_/P) is 12.2.

The experiment site was located in the artificial shelterbelt forest on the northern edge of Minqin Oasis. The shelterbelt was built in 2005 and the average coverage and stand density at the time of this study were 25% and 416 plants per hectare, respectively. Plant heights ranged from 1.5 to 2.5 m. There is no irrigation in the shelterbelt forest. The dominant species is *T*. *ramosissima*, an inverted cone-shaped canopy with no trunk and multiple branches that spread obliquely from the base (Figure 1). The understory vegetation comprises *Nitraria tangutorum*, *Reaumurta soongorica*, and *Kalidium foliatum* and the soil texture is loam.

### Stem Sap Flow Measurements

We measured the SSF of *T*. *ramosissima* during the growing season from May to mid-October in 2020. The SSF gauges (Flow 32, Dynamic Incorporation, Houston, TX, United States) work based on the energy balance method and were used to measure the SSF. We used the stem diameter to select appropriate stems for measuring the SSF. Before the gauges were setup, the mean and range of stem diameters were determined from measurements of 216 stems from 25 *T*. *ramosissima*. Six stems were selected for measuring the SSF from 5 *T*. *ramosissima* (Table 1). We deployed the six SSF gauges that were not all the same model: two SGEX9 gauges, designed for 11 mm stems; a SGEX13 gauge, designed for 14 mm stems; a SGEX13 gauge, designed for 18 mm stems; a SGEX19 gauge, designed for 22 mm stems; and a SGEX25 gauge, designed for 31 mm stems. The range of stem diameters that can be measured by the gauges is 10.2–31.2 mm, which includes more than 93% of *T*. *ramosissima* stem diameters. Measurements from the selected stems can, therefore, provide the representative SSF characteristics for *T*. *ramosissima.*

**TABLE 1:** Characteristics of selected stems of *Tamarix ramosissima* (*T*. *ramosissima*).

Stem number	1	2	3	4	5	6
Canopy (m^2^)	0.45	0.63	0.76	1.26	1.48	1.98
Height (cm)	124	145	152	157	165	178
Stem diameter (mm)	10.2	14.3	11.8	18.8	22.0	31.2

The six selected *T*. *ramosissima* stems grew well throughout the growing period and could support the weight of the sap flow gauge that was used to measure the SSF. We smoothed the selected stems to remove superficial dead skin and attached the gauges to the stems at least 20 cm above the soil surface. To prevent the sensors adhering to the *T*. *ramosissima* branches, natural oil and G4 complexes were smeared on the trunk at the installation positions and on the sensors. Each gauge was protected from the weather by using a shield wrapping of several layers of aluminum foil to prevent solar heating. Once the sensors were installed, the heater input voltage was adjusted according to the operation manual. The gauge readings were recorded at 10 s intervals and stored as a 30-min mean value by using the CR1000 datalogger (Campbell Scientific Incorporation, Logan, UT, United States). The SSF (g⋅h^–1^) can be quantified by using the following equation (Baker and Vanbavel, 1987; Wang D. et al., 2020):


(1) $$SSF=\frac{P_{in}-Q_{v}-Q_{r}}{C_{p\times }dT_{sap}}$$

where *P*_*in*_ is the heater power input (W), *Q*_*v*_ is the upward and downward heat loss along the stem (W), *Q*_*r*_ is the radial heat loss (W), *C*_*p*_ is the specific heat of water (J⋅g^–1^K^–1^), and *dT*_*sap*_ is the temperature differential between the heater and stem section (°C).

### Environmental Factors and Groundwater Measurements

An automatic weather station was established at the study site to monitor micrometeorological factors. The TE525 Bucket Rain Gauge (Texas Electronics Incorporation, Dallas, TX, United States) was used to record precipitation. The Ta and relative humidity (RH) (HC2S3, Campbell Scientific Incorporation, Logan, UT, United States) were measured at 2.7 m above the soil surface and the wind speed and direction (WindSonic75, Gill Instruments Limited, Poole, United Kingdom) were measured at 4.7 m above the soil surface. The net solar radiation and photosynthetically active radiation (PAR) were monitored at 2.5 m above the soil surface by using a radiometer (CNR4) (Kipp & Zonen Incorporation, Delft, Netherlands) and a photosynthetically active radiation quantum sensor (PQS1) (Beijing Truwel Instruments Incorporation, Beijing, China). The soil heat flux was monitored by using a soil heat flux (HFP01) (Hukseflux Incorporation, Delft, Netherlands). All the data were recorded at 10 s intervals and stored as 10 min averages by using the CR3000 datalogger (Campbell Scientific Incorporation, Logan, UT, United States). The vapor pressure deficit (VPD) was calculated from RH and Ta (Cambell and Norman, 1998):


(2) $$VPD=\left(1-RH\right)\times 0.6108\times exp^{\left(\frac{17.27\times Ta}{T+273.3}\right)}$$

where RH is the relative humidity and Ta is the air temperature.

Five volumetric soil moisture profiles were monitored at the study site and all the 0.5 m from the base of the *T*. *ramosissima* selected for the SSF measurements. The five TM soil probes (Decagon Devices Incorporation, Hopkins, United States) measured the soil water content at 5, 10, 20, 40, 80, and 160 cm below the soil surface. The data were recorded at 10 s intervals and stored as 10 min average values by using the CR3000 datalogger (Campbell Scientific Incorporation, Logan, UT, United States) and were calibrated each month by using the gravimetric method. Soil relative extractable water (REW) was calculated as follows (Granier, 1987):


(3) $$REW=\frac{\theta -\theta_{wp}}{\theta_{fc}-\theta_{wp}}$$

where *θ* is the monitored volumetric soil water content and *θ*_*fc*_ (m^3^⋅m^–3^) and *θ*_*wp*_ (m^3^⋅m^–3^) are the field capacity and wilting point, respectively. *θ*_*fc*_ and *θ*_*wp*_ were derived from the soil water characteristic curve. A REW of 0.4 was considered to be the threshold below which there was a soil water deficit.

The groundwater table was monitored at a well 50 m from the experiment site. The groundwater depth was automatically recorded every 30 s and stored as 30 min average values by using a MiniDiver (DI501, Eijkelkamp, Giesbeek, Netherlands).

### Reference ET_0_

Reference ET_0_ (mm⋅d^–1^) was calculated from the Penman–Monteith equation (FAO 56) (Allen et al., 1998):


(4) $$ET_{o}=\frac{0.408\text{Δ}\left(R_{n}-G\right)+\gamma \left(\frac{900}{T_{a}+273}\right)u_{2}\left(e_{s}-e_{a}\right)}{\text{Δ}+\gamma \left(1+0.34u_{2}\right)}$$

where Δ is the slope of the saturation vapor pressure curve (kPa⋅°C^–1^), *e*_*s*_ is the temperature-dependent saturation vapor pressure (kPa), *R*_*n*_ is the net radiation (MJ⋅m^–2^ d^–1^), *G* is the soil heat flux (MJ⋅m^–2^ d^–1^), *e*_*s*_ – *e*_*a*_ is the VPD (kPa), γ is the psychometric constant at temperature (kPa⋅°C^–1^), and *T*_*a*_ (°C) and *u*_2_ (m⋅s^–1^) are the temperature and wind speed, measured 2 m above the soil surface.

### Root Biomass Distribution

We used the trenching method to investigate root biomass and distribution of *T*. *ramosissima* (Komiyama et al., 1987). In the sampling plot, a trench (1 m wide × 1.5 m long × 3.5 m deep) with the 0.1 m distance from the base of *T*. *ramosissima* was excavated to investigate the root distribution of *T*. *ramosissima.* Four replicate trenches were built to collect the samples. Three replicate soil samples (4,000 cm^3^) were collected at 10 cm intervals to 3.5 m depth along one wall of each trench by using metal square corners (20 cm wide × 20 cm long × 10 cm deep). These samples were transported to the laboratory in plastic bags. The fine roots (<2 mm diameter) were separated from the soil by washing through a 0.5-mm sieve. Roots with diameter of <2 mm were defined as feeder roots for water and mineral uptake (Wang et al., 2015; Zhou et al., 2016). Roots were then stored in paper bags until oven-drying at 60°C for 48 h to a constant weight and roots biomass was calculated by a volume basis (kg⋅m^–3^).

### Isotope Sample Collection and Analysis

Isotope samples were collected in May, August, and October 2020. Each rainwater sample was collected immediately after a rain event to avoid evaporation losses. Groundwater samples were collected from a well 50 m from the sample plot. The rainwater and groundwater samples were put into 50 ml sampling bottles and sealed with Parafilm. To reduce the disturbance on *T. ramosissima* transpiration measurement, we collected the xylem samples from six *T*. *ramosissima* that was 30 m away from the experiment site where the *T*. *ramosissima* SSF measurements were made. To avoid oxygen isotope fractionation in the xylem samples, six suberized twigs were collected (diameter: 0.5–0.8 cm; length: 3.0–5.0 cm), scraped with a knife to remove dead skin, and then put into a sampling flask and sealed with Parafilm. Soil samples were collected around 0.5 m from the sampled plants at depths of 10, 20, 30, 40, 50, 60, 80, 100, 120, 160, 200, 250, 300, 350, and 400 cm below the surface by using a 10-cm diameter auger. The collected soil samples were quickly placed into a 50-ml centrifuge tube and sealed with Parafilm. Five soil cores were taken at the site. The collected xylem, soil, and groundwater samples were stored in a refrigerator at –4°C before moisture extraction. Water was extracted from the soil and xylem samples by using a cryogenic vacuum distillation (Li-2000, Lica United Technology Limited, Beijing, China). The isotopic composition of the extracted water was analyzed by using an isotope ratio mass spectrometry iquid water isotope analyzer (LWIA, 912-0008-1001, LOS Gatos Research Incorporation, Mountain View, CA, United States). Due to the fraction of hydrogen when water moves in plants in arid areas (Ellsworth and Williams, 2007), oxygen isotopes were used to analyze water sources for the *T. ramosissima* in this study. The isotope composition (δ^18^O) was calculated as follows:


(5) $$\delta =\frac{R_{sample}}{R_{standard}}-1\times 1000\text{‰}$$

where *R*_*sample*_ and *R*_*standard*_ are the ^18^O/^16^O molar ratios for the sample and for standard water vienna standard mean ocean water (v-SMOW), respectively. The analytical precision was <0.1‰ for δ^18^O.

### Data Analysis

Soil moisture at 0–40 cm depth responds more quickly to precipitation than deeper soil moisture levels. Soil moisture at 40–160 cm depth has a delayed response to heavy precipitation and generally remains stable. We, therefore, divided the soil samples into shallow, middle, and deep layers, corresponding to depths of 0–40, 40–160, and 160–400 cm, respectively.

The correlation analysis and linear regression fitting were used to explore potential environmental controls for the SSF by using SPSS software version 21.0 (SPSS Incorporation, Chicago, IL, United States). The IsoSource model is a mixing model that is designed to partition a large number of sources^1^. We used this to quantify the relative contribution of each water source, based on the isotope mass balance, by using details supplied in Phillips and Gregg (2003).

## Results

### Daily Variations of Environmental Factors

**FIGURE 2:**
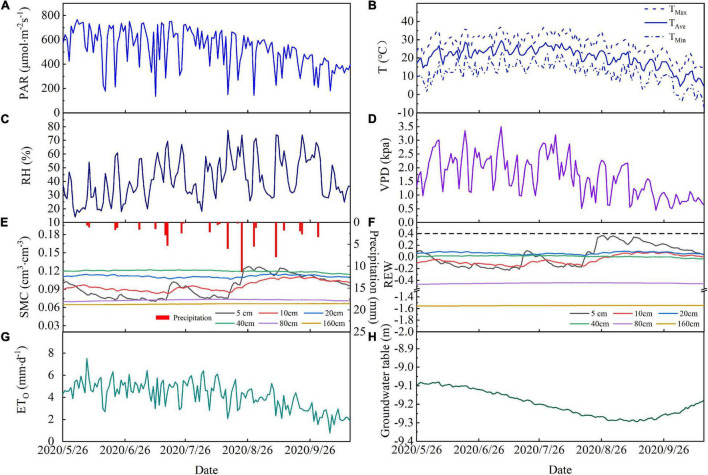
Temporal variations in **(A)** photosynthetically active radiation (PAR), **(B)** average air temperature (Ta_Ave_), maximum air temperature (Ta_Max_), and minimum air temperature (Ta_Min_), **(C)** relative humidity (RH), **(D)** vapor pressure deficit (VPD), **(E)** precipitation and soil moisture, **(F)** soil relative extractable water, **(G)** reference evapotranspiration, and **(H)** groundwater depth during the study period in 2020. Data are plotted at daily resolution.

Daily variations in PAR, Ta, RH, and VPD for May to October in 2020 are shown in Figure 2 and show a clear seasonality for PAR, Ta, RH, and VPD over the study period. Daily PAR, Ta, RH, and VPD ranged from 137.2 to 765.7 μmol⋅m^–2^ s^–1^, 4.2 to 29.6°C, 13.2 to 76.5%, and 0.45 to 3.49 kPa, respectively, during the study period. The highest monthly mean values for PAR, Ta, and VPD (610.99 μmol⋅m^–2^ s^–1^, 24.8°C, and 2.24 kPa) occurred in June or July.

There were 24 precipitation events during the study period, which produced a total rainfall of 63.8 mm. The total event precipitation was less than 5 mm for 19 of these and these low rainfall events resulted in a cumulative total precipitation of 28.8 mm, which is 44% of the total precipitation (Figure 2E). Precipitation exceeded 5 mm for five events and cumulative precipitation from these was 36 mm, which is 56% of the total precipitation. We found that the precipitation events with less than 5 mm of rainfall did not strongly affect soil moisture. Precipitation events with more than 5 mm of rainfall increased the soil moisture at 5 and 10 cm depth, but soil moisture at depths of 20 and 40 cm was less strongly affected. The maximum precipitation event (11.3 mm) induced a rapid increase in soil moisture at 5 and 10 cm depths, which reached their maxima for the observation period following this event, but the REW remained below 0.4 (Figure 2F) at these depths and remained unchanged at other soil depths.

The depth of the groundwater table fluctuated with farmland irrigation and ecological water conveyance (Figure 2H). The depth of the groundwater table remained stable from May to June in 2020 and declined between June and September as groundwater was extracted for irrigation. The groundwater table depth reached its lowest value of 9.3 m on September 13. Ecological water conveyance to the lower reaches of the Shiyang River meant that the groundwater table increased between September and December.

The cumulative ET_0_ was 596.7 mm and daily ET_0_ ranged from 0.8 to 7.5 mm⋅d^–1^, with an average of 4.1 mm⋅d^–1^ during the experiment period (Figure 2G). ET_0_ increased rapidly at the end of May and this increase continued into the summer. From the end of August, ET_0_ decreased steadily (Figure 2G).

### Daily Variation of the Stem Sap Flow

**FIGURE 3:**
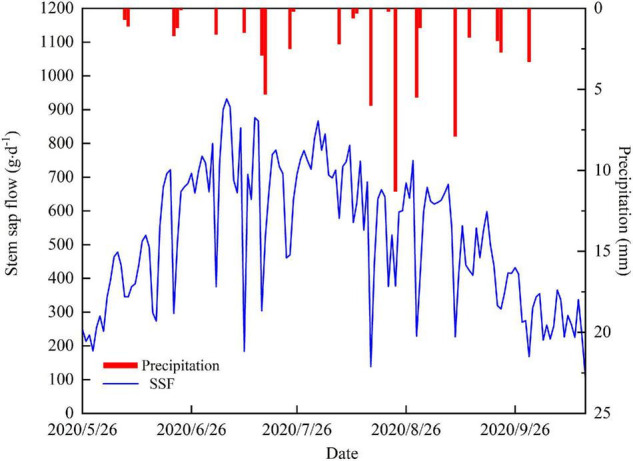
Daily variations in the SSF and precipitation.

The temporal variability of the daily SSF is shown in Figure 3. The average daily SSF for *T*. *ramosissima* during the experiment period was 523.8 g⋅d^–1^. The increase in SSF at the beginning of the growing season coincided with a period of rapid leaf expansion. The SSF remained relatively constant after full leaf expansion. The maximum SSF value was 931.8 g⋅d^–1^ and occurred on July 6. From August, late summer, to the end of the growing season, the SSF showed a clear decreasing trend. It is also notable that the SSF is lower on rainy days than on non-rainfall days.

### Relationships Between the Stem Sap Flow and Environmental Factors

**FIGURE 4:**
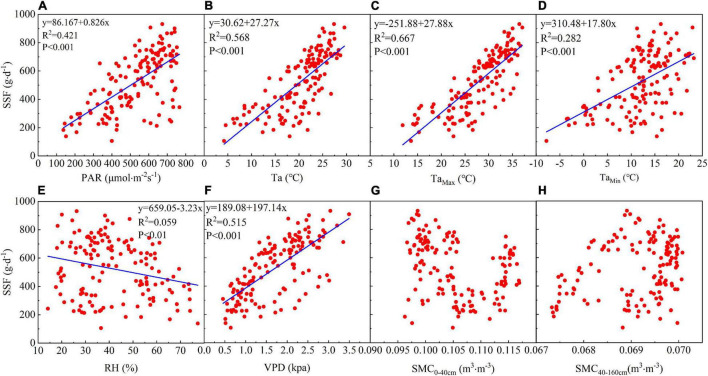
The relationship between the daily SSF and eight environmental factors: **(A)** PAR, **(B)** Ta_Ave_, **(C)** Ta_Max_, **(D)** Ta_Min_, **(E)** RH, **(F)** VPD, **(G)** soil moisture content_0–40 cm_ (SMC_0–40 cm_), and **(H)** soil moisture content_40–160 cm_ (SMC_40–160 cm_) during the growing season in 2020.

Linear regression analysis showed that the daily SSF is strongly correlated with meteorological factors. There is a significant positive linear correlation between the daily SSF and PAR and PAR explains 42.7% of the SSF variance (Figure 4A). The daily SSF data are significantly positively linearly correlated with Ta, and particularly with Ta_max_, which explains 65.2% of the SSF variance (Figures 4B–D). The daily SSF is significantly correlated with VPD, which explains 51.5% of the SSF variance (Figure 4F). The SSF data for *T. ramosissima* are negatively correlated with RH (Figure 4E).

There is no significant correlation between the daily SSF and soil moisture in the shallow (0–40 cm) and middle soil layers (40–160 cm). Figures 4G,H shows that there is no significant correlation between the SSF and θ_0–40 cm_ or θ_40–160 cm_, indicating that soil moisture may not have been the main factor affecting the SSF during the growing season in 2020.

**FIGURE 5:**
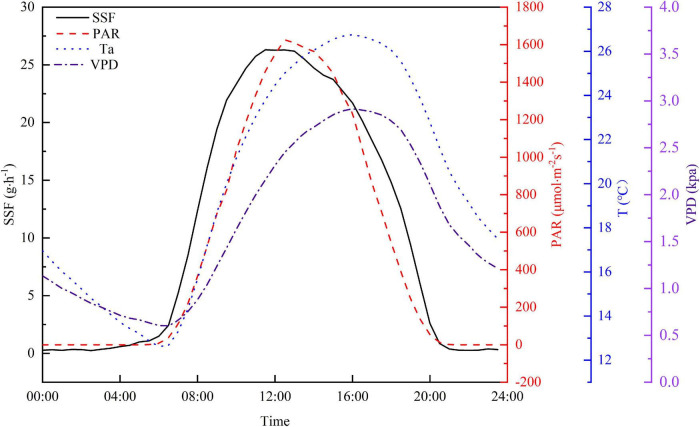
Average diurnal cycle for half-hourly averaged SSF, PAR, Ta, and VPD during the growing season in 2020.

Diurnal variations in PAR, Ta, VPD, and SSF are shown in Figure 5 to further explore relationships between the SSF and these meteorological factors. The half-hourly averaged values from the experiment period were used in this study to minimize the uncertainty. The SSF increased sharply after sunrise and this preceded the increase in PAR by approximately 1.5 h. Both the Ta and VPD rose rapidly after sunrise. The increase in Ta and VPD lagged behind the increase in PAR and the peak value for both occurred 3 h after the PAR peak. Although the shapes of the diurnal curves are similar for the four variables, the time of the day for peak values is different. The peak time for PAR lagged behind that for the SSF by approximately 1.5 h and the PAR peak is narrower than the SSF peak. The Ta and VPD peaks occurred at almost the same time, but lagged behind the SSF peak by about 4.5 h. The SSF decreased to a relatively low level after sunset and then remained fairly constant, with a curve that remained ahead of the PAR curve by a factor of 1 h. In contrast, Ta and VPD decreased steadily until sunrise on the following day.

**FIGURE 6:**
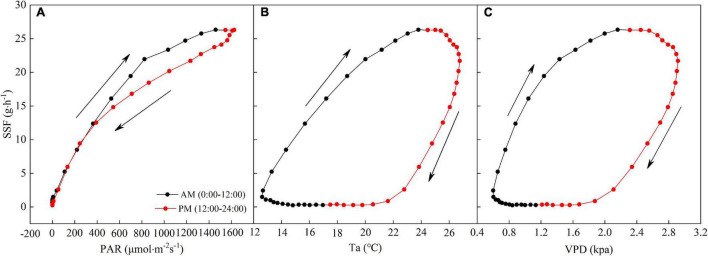
Average relationships between half-hourly averaged SSF and **(A)** PAR, **(B)** Ta, and **(C)** VPD during a day. The arrows indicate the direction of rotation.

The diurnal hysteresis loops for the SSF and PAR and Ta and VPD are shown in Figure 6. The arrows in Figure 6 denote the rotation directions of the hysteresis loops. Hysteresis loops with clockwise rotations were observed for the averaged relationships between the SSF and PAR and Ta and VPD in a day, indicating that variations in PAR, Ta, and VPD lagged behind those in the SSF.

### Soil Moisture Characteristics at 0–400 cm Depth and Water Sources for *Tamarix ramosissima*

**FIGURE 7:**
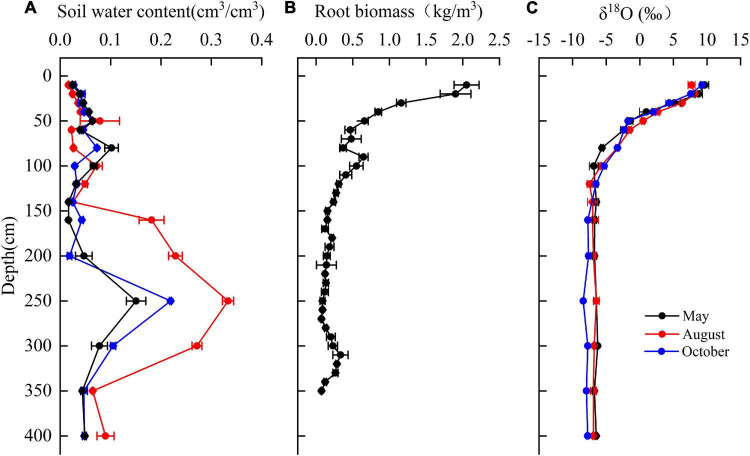
The variation of **(A)** soil water content, **(B)** fine root biomass of *T*. *ramosissima* (0–350 cm), and **(C)** δ^18^O in the vertical soil profile during the 2020 growing season.

The mean soil moisture was 0.06 cm^3^⋅cm^–3^ over depths of 0–400 cm. Soil moisture varied with depth and with the sampling time (Figure 7A). At 0–80 cm, soil moisture increased with an increasing depth. Soil moisture increased sharply at 140–250 cm depth, where the highest clay content was encountered and decreased over 250–400 cm depth.

The root biomass (<2 mm) was observed up to 350 cm depth with approximately 66% of fine root biomass at the depth of 0–100 cm (Figure 7B) and 43% of fine root concentrated in the shallow soil layers (0–40 cm). The fine root biomass in middle (40–160 cm) and deep soil layers (160–350 cm) accounted for 33 and 22% of total fine root biomass, respectively.

The δ^18^O values for soil moisture that was sampled at different depths and at different observation times are shown in Figure 7C. The mean δ^18^O values for soil water in the shallow soil layers that were sampled in May, August, and October are 6.1341, 6.2017, and 5.789‰, respectively. These values are significantly higher than the values for other soil layers (*P* < 0.05). The δ^18^O values for soil moisture in the middle soil layer decreased continuously with depth and exhibited moderate seasonality. The mean values for this soil layer are –5.16077, –4.51675, and –4.4872‰ for May, August, and October, respectively. The δ^18^O values for deep soil moisture were relatively stable and exhibited no significant seasonality.

The δ^18^O values for the groundwater samples did not vary significantly in May, August, and October and the mean value for these months was –6.92‰ (Table 2). The δ^18^O values for the xylem that was sampled from *T. ramosissima* in May, August, and October were –6.32, –6.80477, and –7.1041‰, respectively. The oxygen isotope values for the xylem water samples were close to the values for the deep soil moisture (160–400 cm) and groundwater (910 cm) samples, indicating that the xylem water was mostly absorbed from moisture in deep soil layers and from groundwater.

**TABLE 2:** The oxygen isotopic values for xylem and potential water sources.

δ^18^O	May	August	October
Xylem water (‰)	–6.3233	–6.80477	–7.1041
Shallow soil water (‰)	6.1341	6.2017	5.798
Middle soil water (‰)	–5.16077	–4.51675	–4.4872
Deep soil water (‰)	–6.30665	–6.7867	–7.0936
Groundwater (‰)	–6.5167	–6.9358	–7.3166

**FIGURE 8:**
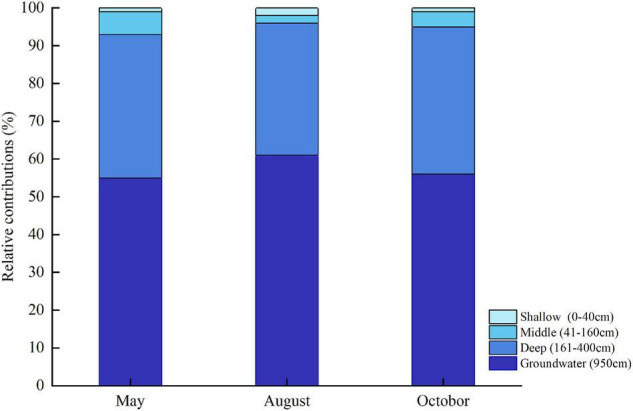
Water uptake from shallow, middle, and deep soil layers and from groundwater for *T. ramosissima* during the 2020 growing season.

The IsoSource model showed that deep soil moisture and groundwater were the main water sources for the *T. ramosissima* (Figure 8). In May, August, and October, the relative combined contributions of deep soil moisture and groundwater to the total *T. ramosissima* water intake were 93, 96, and 95%, respectively. *T. ramosissima* uptake water from the shallow and middle soil layers was very low in May, August, and October.

## Discussion

### Characteristics of *Tamarix ramosissima* Stem Sap Flow

The results presented here demonstrate that the cumulative and daily SSF for *T. ramosissima* was very low during the experiment period. Over the whole growing season, the cumulative SSF and the average daily SSF are lower than the SSF values that have been reported for *T. ramosissima* in other regions (Li et al., 2018; Chen et al., 2019). Xu et al. (2009) used the same methods as this study and found that the mean daily SSFs for *T. ramosissima* with stem diameters of 3.5 and 2.0 cm in the Taklamakan Desert region were 6.322 and 1.179 kg, respectively, representing 12.1 and 2.4 times the results found in this study, respectively. This large difference may be explained by differences in irrigation. In the Taklamakan Desert region, *T. ramosissima* depends heavily on artificial irrigation from water wells and soil moisture levels are relatively high (Xu et al., 2009). In contrast, the artificial shelterbelt in this study area is in a natural region, without irrigation. An additional explanation for the low SSF found at the Minqin site may be that the trees are older, which is suggested by the lower vigor of the trees here. Research has shown that plant age leads to differences in photosynthetic rate, leaf area, stomatal conductance, and SSF (Ryan et al., 2006; Forrester et al., 2010). Younger plants grow faster and use more water per unit leaf area than older plants (Medeiros and Pockman, 2010). *T. ramosissima* in the Taklamakan Desert region is 8 years old (Xu et al., 2009), while *T. ramosissima* in this study region is 15 years old and, therefore, more mature and less vigorous, which may contribute to the lower SSF values.

### Influence of Environmental Factors on the Stem Sap Flow

Plant transpiration is limited by the available energy and by evaporative demand (Jarvis, 1976; Schulze et al., 1994; Zha et al., 2017). Thus, the SSF is directly influenced by meteorological factors. PAR is the part of the incident solar radiation that is used by plants for photosynthesis and the SSF is closely related to photosynthesis (Bovard et al., 2005; Clausnitzer et al., 2011; Tie et al., 2017). PAR, Ta, and VPD explain 42.7, 65.2, and 51.5% of the daily variance in the SSF, respectively. Our results show that PAR, Ta, and VPD are the controlling factors for the daily SSF. The main reason for this is that Ta and VPD increase with an increasing PAR, which induces an increase in the potential difference across the leaf-to-air water boundary. This conclusion is similar to that in previous studies (Bovard et al., 2005; Shen et al., 2015; Tie et al., 2017; Zhang Q. Y. et al., 2018).

Plant transpiration is conducted via stomatal processes and the hysteresis between environmental factors and the SSF can be regarded as the adaptation of plants to environmental factors caused by water limitation (Matheny et al., 2014; Ma et al., 2017; Zha et al., 2017; Hong et al., 2019; Wang et al., 2019). Our results show that the daily cycle for PAR, Ta, and VPD (24 h) lags behind that for the SSF (Figure 6), which is in agreement with previous studies (Ma et al., 2017; Hong et al., 2019; Wang et al., 2019). Generally, the maximum PAR occurs at noon, while the maximum temperature and the maximum VPD usually appear in the afternoon. In the morning, as atmospheric evaporation demand (PAR, Ta, and VPD) increases, the SSF gradually increases. As the SSF increases, the transpiration rate of *T. ramosissima* increases until it exceeds the supply of soil moisture. When PAR, Ta, and VPD reach their peaks, plants close some stomata, as a form of self-protection, to prevent excessive water extraction from the trunk. Failure to do this could result in xylem vessel embolism and the collapse of the hydrological conductive system of the xylem (Zeppel et al., 2004; Chen et al., 2011).

Previous studies have shown that the relationship between the SSF and soil water content is not consistent. For example, some studies have shown that soil moisture is the dominant controlling factor for the SSF (Shen et al., 2015; Ma et al., 2017; Zha et al., 2017). Our results show that, in this study, there was no significant correlation between the SSF and soil moisture in shallow (0–40 cm) and middle (40–160 cm) soil layers during the growing season, which is consistent with previous findings (Hölscher et al., 2005; Horna et al., 2011; Jiao et al., 2016; Tie et al., 2017). The relatively low soil water content in the shallow (0–40 cm) and middle soil layers (40–160 cm) means that there is no significant relationship between the SSF and soil moisture in these soil layers. Desert plants do not respond directly to rainfall, but rather respond to the availability of soil water (Reynolds et al., 2004; Robertson et al., 2009). In water-limited regions, most precipitation occurs in smaller precipitation events (resulting in less than 5 mm rainfall), which moisten the uppermost soil layers. Soil moisture is readily and directly lost to the atmosphere from these layers through evaporation (Beatley, 1974; Dougherty et al., 1996). Heavy precipitation events (resulting in more than 10 mm rainfall) occur less frequently in arid areas, but serve to recharge moisture levels in deep soil layers and so increase the plant SSF (Schwinning and Sala, 2004; Cheng et al., 2006). Our results show that the soil water content in the shallow and middle soil layers ranged from 0.07 to 0.12 cm^3^⋅cm^–3^ and from 0.06 to 0.07 cm^3^⋅cm^–3^ during the experimental period, respectively. As shown in Figures 2C,D, smaller rainfall events had little effect on soil moisture and the soil REW was less than 0.4 throughout the experiment period. Similar to this, Hayat et al. (2020) found that when the soil moisture was below a threshold, the transpiration rate of *Salix psammophila* did not increase with increase in the soil moisture content.

Our data show that *T. ramosissima* SSF was higher on sunny days than on rainy days and the greater the rainfall, the lower the SSF. This may be due to precipitation-driven changes in PAR, Ta, VPD, and RH. The range and thickness of clouds are greater on rainfall days than on non-rainfall days and this reduces PAR and Ta. Rainfall events also lead to an increase in RH, which can contribute to the saturation of leaf surfaces and to crease in leaf turgor (Zhao and Liu, 2010; Zha et al., 2017). In addition, stomata tend to close when environmental conditions change and leaf water status may be responsible for the decrease in the SSF during rainfall events (Granier, 1987; Irvine et al., 1998). Wang D. et al. (2020) illustrated that the SSF was much lower on high rainfall days than on sunny and smaller rainfall days for *Caragana korshinskii* and *Salix psammophila*. The reason for this is that greater precipitation depth and longer duration leave the vegetation canopy in a humid state for a long time and since solar radiation is mainly used to evaporate water from the vegetation canopy (Kume et al., 2006), this leads to a longer stomatal closure and a decrease in the SSF.

### Water Sources for *Tamarix ramosissima* Transpiration

The importance of groundwater and moisture levels in deep soil layers for plant transpiration in the arid region has been reported (Cui et al., 2010; Huang et al., 2015). To maintain transpiration when the moisture content of the shallow and middle soil layers is extremely low, *T. ramosissima* needs to take-up water from deep soil layers and groundwater. Although the δ^18^O values for *T. ramosissima* xylem water have a slight seasonality, deep soil water and groundwater are the main water sources, which are a finding that are consistent with previous studies (Chen et al., 2017; Li et al., 2019; Dong et al., 2020). In generally, water sources for plants are affected by soil water conditions and by plant root systems (Asbjornsen et al., 2007; Dong et al., 2020). *T. ramosissima* has a well-developed root system: lateral roots (diameter < 2 mm) are mainly distributed through 0–40 cm soil depth, allowing them to fully absorb shallow soil moisture. However, the scarcity and variability of rainfall and the strong evaporation from the upper soil layers make moisture levels at shallow depths extremely low. Studies have demonstrated that when soil moisture is below a threshold, the lateral, absorbing roots in the upper soil layers are a state of dormancy and plants are more likely to use deep soil water and groundwater (Ehleringer and Dawson, 1992; Flanagan et al., 1992; Xu and Li, 2006). *T. ramosissima* has very deep roots that can reach 10 m belowground (Yu et al., 2013; Dong et al., 2020). Plants may recharge groundwater to deep soil layers via root hydraulic redistribution in arid areas (Guo et al., 2012; Yu et al., 2013). These characteristics make it possible for *T. ramosissima* to readily take-up groundwater and deep soil water.

### Water Use Strategies of *Tamarix ramosissima*

Due to low rainfall, high atmospheric evaporative demand, and a deep groundwater table, most desert shrubs experience severe water shortages during their life cycle (Xu and Li, 2006). Based on basic plant physiology, we might expect precipitation to strongly impact on the transpiration of *T. ramosissima*. In this study, *T. ramosissima* transpiration increases significantly in response to heavy rainfall (Figure 3), probably because the rainfall resulted in only an insignificant increase in soil water (Figure 2F). The root distribution of *T. ramosissima* means that it can access groundwater to support its subsistence during the dry period by using deep roots (Yu et al., 2013; Gou and Miller, 2014; Dong et al., 2020). This is called the water acquisition strategy of the plant (Brouillette et al., 2013). The water uptake pattern of the plant is controlled by resistance (rhizosphere resistance and root resistance) and by the potential gradient within the soil-plant-atmosphere continuum (SPAC). In general, the uptake of groundwater requires extra energy to overcome gravitational pressure and uptake from soil water is generally more favorable than from groundwater during the wet period. However, the increase in rhizosphere resistance and root resistance, which are attributable to the strong decline in soil water potential, exert a remarkable influence on water uptake (Gou and Miller, 2014). The influence of gravity on water uptake was insignificantly compared with the strong decline in soil water potential during the dry period. The greater energy costs for groundwater uptake in regions with a deep groundwater table (9.1 m) mean that using this water source only allows the plant to survive, not necessarily to flourish (Hacke et al., 2000) and the plant still suffers from water stress even though the roots can access groundwater.

Plants may adapt to water limitations with water-conservation strategies (Brouillette et al., 2013). Desert shrubs reduce their water consumption by closing their stomata when exposed to dehydration stresses. At this study site, the dry period was accompanied by high VPD during the growing season, which probably led to a decreased stomatal conductance and more significant control of the stomatal conductance on the SSF of *T. ramosissima*. The water supply was generally insufficient to balance the high *T. ramosissima* transpiration demand at mid-day, resulting in a clockwise hysteresis loop. The hysteresis effect reflects plant acclimatization to water limitations, owing to stomatal conductance being inherently decided by plant hydrodynamics (Matheny et al., 2014).

## Conclusion

Understanding the relationship between plant transpiration and the ecosystem water supply is a necessary for managing the limited water resources in arid regions. The SSF of *T. ramosissima* has a clear seasonality. Due to water scarcity in the shallow and middle soil layers, *T. ramosissima* mainly uptake water from deep soil layers and from groundwater. PAR, Ta, and VPD are the controlling factors for *T. ramosissima* SSF.

This study also sheds light on that groundwater and deep soil water play an important role in ecological development in water-limited regions. For sustainable development, future afforestation efforts should evaluate the carrying capacity of groundwater. Drought-enduring native plants and a suitable planting density should be considered when planning revegetation initiatives, so as to prevent excessive consumption of deep soil water and groundwater.

## Data Availability Statement

The raw data supporting the conclusions of this article will be made available by the authors, without undue reservation.

## Author Contributions

FL and QY performed the experiments. QY contributed technical advice in equipment installation. FL contributed to the collection of the data and writing the first draft. QY, XX, FP, and SM contributed to the writing—reviewing, editing, and supervision. JP, YS, and XC provided assistance during data processing and data analysis. All authors contributed to the article and approved the submitted version.

## Conflict of Interest

The authors declare that the research was conducted in the absence of any commercial or financial relationships that could be construed as a potential conflict of interest.

## Publisher’s Note

All claims expressed in this article are solely those of the authors and do not necessarily represent those of their affiliated organizations, or those of the publisher, the editors and the reviewers. Any product that may be evaluated in this article, or claim that may be made by its manufacturer, is not guaranteed or endorsed by the publisher.

## Footnotes

1
http://www.epa.gov/wed/pages/models/stableIsotopes/isosource/isosource.htm

## References

[B1] Allen R. Pereira L. Raes D. Smith M. Allen R. G. Pereira L. S. (1998). Crop Evapotranspiration: guidelines for Computing Crop Water Requirements, FAO Irrigation and Drainage. *J. Hydrol.* 285 19–40.

[B2] Asbjornsen H. Mora G. Helmers M. J. (2007). Variation in water uptake dynamics among contrasting agricultural and native plant communities in the Midwestern U.S. *Agric. Ecosyst. Environ.* 121 343–356. 10.1016/j.agee.2006.11.009

[B3] Baker J. M. Vanbavel C. H. M. (1987). Measurement of mass-flow of water in the stems of herbaceous plants. *Plant Cell Environ.* 10 777–782.

[B4] Beatley J. C. (1974). Phenological events and their environmental triggers in mojave-desert ecosystems. *Ecology* 55 856–863. 10.2307/1934421

[B5] Bovard B. D. Curtis P. S. Vogel C. S. Su H. B. Schmid H. P. (2005). Environmental controls on sap flow in a northern hardwood forest. *Tree Physiol.* 25 31–38. 10.1093/treephys/25.1.31 15519983

[B6] Brouillette L. C. Mason C. M. Shirk R. Y. Donovan L. A. (2013). Adaptive differentiation of traits related to resource use in a desert annual along a resource gradient. *New Phytol.* 201 1316–1327. 10.1111/nph.12628 24325125

[B7] Cambell G. S. Norman J. M. (1998). *An Introduction to Environmental Biophysics.* New York, NY: Springer.

[B8] Cassiani G. Boaga J. Vanella D. Perri M. T. Consoli S. (2015). Monitoring and modelling of soil-plant interactions: the joint use of ERT, sap flow and eddy covariance data to characterize the volume of an orange tree root zone. *Hydrol. Earth Sys. Sci.* 19 2213–2225. 10.5194/hess-19-2213-2015

[B9] Chen H. Yang C. Ren A. Guo K. Feng X. Li J. (2019). The Evapotranspiration of Tamarix and Its Response to Environmental Factors in Coastal Saline Land of China. *Water* 11:2273. 10.3390/w11112273

[B10] Chen L. Zhang Z. Li Z. Tang J. Caldwell P. Zhang W. (2011). Biophysical control of whole tree transpiration under an urban environment in Northern China. *J. Hydrol.* 402 388–400. 10.1016/j.jhydrol.2011.03.034

[B11] Chen Y. Li W. Zhou H. Chen Y. Ma J. Fu A. (2017). Experimental study on water transport observations of desert riparian forests in the lower reaches of the Tarim River in China. *Int. J. Biometeorol.* 61 1055–1062. 10.1007/s00484-016-1285-x 28283759

[B12] Cheng X. An S. Li B. Chen J. Lin G. Liu Y. (2006). Summer rain pulse size and rainwater uptake by three dominant desert plants in a desertified grassland ecosystem in northwestern China. *Plant Ecol.* 184 1–12.

[B13] Chirino E. Bellot J. Sánchez J. R. (2011). Daily sap flow rate as an indicator of drought avoidance mechanisms in five Mediterranean perennial species in semi-arid southeastern Spain. *Trees* 25 593–606. 10.1007/s00468-010-0536-4

[B14] Clausnitzer F. Koestner B. Schwaerzel K. Bernhofer C. (2011). Relationships between canopy transpiration, atmospheric conditions and soil water availability-Analyses of long-term sap-flow measurements in an old Norway spruce forest at the Ore Mountains/Germany. *Agric. Forest Meteorol.* 151 1023–1034. 10.1016/j.agrformet.2011.04.007

[B15] Cochard H. Coll L. Le Roux X. Améglio T. (2002). Unraveling the Effects of Plant Hydraulics on Stomatal Closure during Water Stress in Walnut. *Plant Physiol.* 128 282–290. 10.1104/pp.010400 11788773 PMC148995

[B16] Cui B. Yang Q. Zhang K. Zhao X. You Z. (2010). Responses of saltcedar (Tamarix chinensis) to water table depth and soil salinity in the Yellow River Delta. *China Plant Ecol.* 209 279–290. 10.1007/s11258-010-9723-z

[B17] David T. S. Pinto C. A. Nadezhdina N. Kurz-Besson C. Henriques M. O. Quilho T. (2013). Root functioning, tree water use and hydraulic redistribution in Quercus suber trees: a modeling approach based on root sap flow. *Forest Ecol. Manag.* 307 136–146. 10.1016/j.foreco.2013.07

[B18] Dong Z. (2004). *Research on farmland Wind–Sand Disaster of Oasis and its Contral Mechanism in Ulan Buh Desert.* Beijing: Beijing Forestry University.

[B19] Dong Z. Li S. Zhao Y. Lei J. Wang Y. Li C. (2020). Stable oxygen-hydrogen isotopes reveal water use strategies of Tamarix taklamakanensis in the Taklimakan Desert. *China. J. Arid Land* 12 115–129. 10.1007/s40333-020-0051-4

[B20] Dougherty R. L. Lauenroth W. K. Singh J. S. (1996). Response of a grassland cactus to frequency and size of rainfall events in a North American shortgrass steppe. *J. Ecol.* 84 177–183. 10.2307/2261353

[B21] Ehleringer J. R. Dawson T. E. (1992). Water-uptake by plants - perspectives from stable isotope composition. *Plant Cell Environ.* 15 1073–1082. 10.1111/j.1365-3040.1992.tb01657.x

[B22] Ellsworth P. Z. Williams D. G. (2007). Hydrogen isotope fractionation during water uptake by woody xerophytes. *Plant Soil* 291 93–107. 10.1007/s11104-006-9177-1

[B23] Flanagan L. B. Ehleringer J. R. Marshall J. D. (1992). Differential uptake of summer precipitation among cooccurring trees and shrubs in a pinyon-juniper woodland. *Plant Cell Environ.* 15 831–836. 10.1111/j.1365-3040.1992.tb02150.x

[B24] Forrester D. I. Collopy J. J. Morris J. D. (2010). Transpiration along an age series of Eucalyptus globulus plantations in southeastern Australia. *Forest Ecol. Manag.* 259 1754–1760. 10.1016/j.foreco.2009.04.023

[B25] Fu S. Sun L. Luo Y. (2016). Combining sap flow measurements and modelling to assess water needs in an oasis farmland shelterbelt of Populus simonii Carr in Northwest China. *Agric. Water Manag.* 177 172–180. 10.1016/j.agwat.2016.07.015

[B26] Gao Y. Xia J. Chen Y. Zhao Y. Kong Q. Lang Y. (2016). Effects of extreme soil water stress on photosynthetic efficiency and water consumption characteristics of Tamarix chinensis in China’s Yellow River Delta. *J. Forestry Res.* 28 491–501. 10.1007/s11676-016-0339-6

[B27] Good S. P. Noone D. Bowen G. (2015). Hydrologic connectivity constrains partitioning of global terrestrial water fluxes. *Science* 349 175–177. 10.1126/science.aaa5931 26160944

[B28] Gou S. Miller G. (2014). A groundwater-soil-plant-atmosphere continuum approach for modelling water stress, uptake, and hydraulic redistribution in phreatophytic vegetation. *Ecohydrology* 7 1029–1041. 10.1002/eco.1427

[B29] Granier A. (1987). Evaluation of transpiration in a douglas-fir stand by means of sap flow measurements. *Tree Physiol.* 3 309–319. 10.1093/treephys/3.4.309 14975915

[B30] Grossiord C. Sevanto S. Borrego I. Chan A. M. Collins A. D. Dickman L. T. (2017). Tree water dynamics in a drying and warming world. *Plant Cell Environ.* 40 1861–1873. 10.1111/pce.12991 28556263

[B31] Guo F. Pei Z. Xue S. Zhuang W. (2012). Change characteristics in soil water content in zone and ecidence of root hydraulic lif in Tamarix Ramosissima thickets on sand dunes. *Chinese J. Plant Ecol.* 36 1033–1042.

[B32] Hacke U. G. Sperry J. S. Ewers B. E. Ellsworth D. S. Schafer K. V. R. Oren R. (2000). Influence of soil porosity on water use in Pinus taeda. *Oecologia* 124 495–505. 10.1007/pl00008875 28308388

[B33] Han C. Chen N. Zhang C. K. Liu Y. J. Khan S. Lu K. L. (2019). Sap flow and responses to meteorological about the Larix principis-rupprechtii plantation in Gansu Xinlong mountain, northwestern China. *Forest Ecol. Manag.* 451:117519. 10.1016/j.foreco.2019.117519

[B34] Hayat M. Zha T. S. Jia X. Iqbal S. Qian D. Bourque C. P. A. (2020). A multiple-temporal scale analysis of biophysical control of sap flow in Salix psammophila growing in a semiarid shrubland ecosystem of northwest China. *Agric. Forest Meteorol.* 288:107985. 10.1016/j.agrformet.2020.107985

[B35] Hölscher D. Koch O. Korn S. Leuschner C. (2005). Sap flux of five co-occurring tree species in a temperate broad-leaved forest during seasonal soil drought. *Trees* 19 628–637. 10.1007/s00468-005-0426-3

[B36] Hong L. Guo J. B. Liu Z. B. Wang Y. H. Ma J. Wang X. (2019). Time-Lag Effect Between Sap Flow and Environmental Factors of Larix principis-rupprechtii Mayr. *Forests* 10:971. 10.3390/f10110971

[B37] Horna V. Schuldt B. Brix S. Leuschner C. (2011). Environment and tree size controlling stem sap flux in a perhumid tropical forest of Central Sulawesi, Indonesia. *Annals. Forest Sci.* 68 1027–1038. 10.1007/s13595-011-0110-2

[B38] Huang J. T. Zhou Y. X. Yin L. H. Wenninger J. Zhang J. Hou G. C. (2015). Climatic controls on sap flow dynamics and used water sources of Salix psammophila in a semi-arid environment in northwest China. *Environ. Earth Sci.* 73 289–301. 10.1007/s12665-014-3505-1

[B39] Irvine J. Perks M. P. Magnani F. Grace J. (1998). The response of Pinus sylvestris to drought: stomatal control of transpiration and hydraulic conductance. *Tree Physiol.* 18 393–402. 12651364 10.1093/treephys/18.6.393

[B40] Jarvis P. G. (1976). Interpretation of variations in leaf water potential and stomatal conductance found in canopies in field. *Philosophical Trans. Royal Soc. London Series B Biol. Sci.* 273 593–610. 10.1098/rstb.1976.0035 PMC4360119 25750234

[B41] Jia X. Shao M. A. Zhu Y. Luo Y. (2017). Soil moisture decline due to afforestation across the Loess Plateau, China. *J. Hydrol.* 546 113–122. 10.1016/j.jhydrol.2017.01.011

[B42] Jia Y.-H. Shao M.-A. (2014). Dynamics of deep soil moisture in response to vegetational restoration on the Loess Plateau of China. *J. Hydrol.* 519 523–531. 10.1016/j.jhydrol.2014.07.043

[B43] Jiao L. Lu N. Sun G. Ward E. J. Fu B. (2016). Biophysical controls on canopy transpiration in a black locust (*Robinia pseudoacacia*) plantation on the semi-arid Loess Plateau. *China. Ecohydrol.* 9 1068–1081. 10.1002/eco.1711

[B44] Jin Y. Wang X. Zhang Y. Pan Y. Xu H. Hu R. (2018). Evapotranspiration of xerophytic shrub Salsola passerina and Reaumuria soongorica in an arid desert ecosystem of NW China. *Hydrol. Res.* 49 1847–1863. 10.2166/nh.2018.170

[B45] Johnson J. D. Tognetti R. Paris P. (2002). Water relations and gas exchange in poplar and willow under water stress and elevated atmospheric CO2. *Physiol. Plant.* 115 93–100. 10.1034/j.1399-3054.2002.1150111.x 12010472

[B46] Komiyama A. Ogino K. Aksornkoae S. Sabhasri S. (1987). Root biomass of a mangrove forest in southern thailand .1. estimation by the trench method and the zonal structure of root biomass. *J. Trop. Ecol.* 3 97–108. 10.1017/s0266467400001826

[B47] Kucera J. Brito P. Jimenez M. S. Urban J. (2017). Direct Penman-Monteith parameterization for estimating stomatal conductance and modeling sap flow. *Trees Struct. Funct.* 31 873–885. 10.1007/s00468-016-1513-3

[B48] Kume T. Kuraji K. Yoshifuji N. Morooka T. Sawano S. Chong L. (2006). Estimation of canopy drying time after rainfall using sap flow measurements in an emergent tree in a lowland mixed-dipterocarp forest in Sarawak. *Malaysia Hydrol. Proc.* 20 565–578. 10.1002/hyp.5924

[B49] Lambs L. Berthelot M. (2002). Monitoring of water from the underground to the tree: first results with a new sap extractor on a riparian woodland. *Plant Soil* 241 197–207. 10.1023/a:1016131102232

[B50] Lei J. Wang X. Wang D. (2003). The formation of the blown sand disaster to the Tarim desert highway Xinjiang, China. *Arid Zone res.* 20 1–6.

[B51] Li B. Chen Y. Shi X. Chen Z. Li W. (2012). Temperature and precipitation changes in different environments in the arid region of northwest China. *Theoretical Appl. Climatol.* 112 589–596. 10.1007/s00704-012-0753-4

[B52] Li E. Tong Y. Huang Y. Li X. Wang P. Chen H. (2019). Responses of two desert riparian species to fluctuating groundwater depths in hyperarid areas of Northwest China. *Ecohydrology* 12:e2078. 10.1002/eco.2078

[B53] Li S. Du J. Xiao H. (2018). Dynamics of sap flow rates in stems of typical desert shrub Tamarix ramosissima and its responses to the environmental factors. *J. Arid Land Resour. Environ.* 12 170–175.

[B54] Li S. Xiao H. Cheng Y. Wang F. (2015). Water use measurement by non-irrigated Tamarix ramosissima in arid regions of Northwest China. *Sci. Cold Arid Regions* 7 146–156.

[B55] Ma C. K. Luo Y. Shao M. G. Li X. D. Sun L. Jia X. X. (2017). Environmental controls on sap flow in black locust forest in Loess Plateau, China. *Sci. Rep.* 7:13160. 10.1038/s41598-017-13532-8 29030585 PMC5640688

[B56] Matheny A. M. Bohrer G. Vogel C. S. Morin T. H. He L. Frasson R. P. D. M. (2014). Species-specific transpiration responses to intermediate disturbance in a northern hardwood forest. *Biogeosciences* 119 2292–2311. 10.1002/2014jg002804

[B57] Medeiros J. S. Pockman W. T. (2010). Carbon gain and hydraulic limits on water use differ between size classes of Larrea tridentata. *J. Arid Environ.* 74 1121–1129. 10.1016/j.jaridenv.2010.05.008

[B58] Nippert J. B. Butler J. J. Jr. Kluitenberg G. J. Whittemore D. O. Arnold D. Spal S. E. (2010). Patterns of Tamarix water use during a record drought. *Oecologia* 162 283–292. 10.1007/s00442-009-1455-1 19756759

[B59] Novick K. A. Ficklin D. L. Stoy P. C. Williams C. A. Bohrer G. Oishi A. C. (2016). The increasing importance of atmospheric demand for ecosystem water and carbon fluxes. *Nat. Clim. Change* 6 1023–1027. 10.1038/nclimate3114

[B60] Nyabwisho K. A. Diels J. Kahimba F. C. Van Griensven A. (2020). Measuring soil evaporation from a cropped land in the semi-arid Makanya catchment, Northern Tanzania: methods and challenges. *Phys. Chem. Earth* 118:102884. 10.1016/j.pce.2020.102884

[B61] Pan J. Huang C. Peng F. Zhang W. Luo J. Ma S. (2020). Effect of Arbuscular Mycorrhizal Fungi (AMF) and Plant Growth-Promoting Bacteria (PGPR) Inoculations on Elaeagnus angustifolia L. in Saline Soil. *Appl. Sci.* 10:945. 10.3390/app10030945

[B62] Pan Y.-X. Wang X.-P. Ma X.-Z. Zhang Y.-F. Hu R. (2020). The stable isotopic composition variation characteristics of desert plants and water sources in an artificial revegetation ecosystem in Northwest China. *Catena* 189 104499–104499. 10.1016/j.catena.2020.104499

[B63] Phillips D. L. Gregg J. W. (2003). Source partitioning using stable isotopes: coping with too many sources. *Oecologia* 136 261–269. 12759813 10.1007/s00442-003-1218-3

[B64] Qu Y. Kang S. Li F. Zhang J. Xia G. Li W. (2007). Xylem sap flows of irrigatedTamarix elongata Ledeb and the influence of environmental factors in the desert region of Northwest China. *Hydrol. Proc.* 21 1363–1369. 10.1002/hyp.6314

[B65] Reynolds J. F. Kemp P. R. Ogle K. Fernandez R. J. (2004). Modifying the ‘pulse-reserve’ paradigm for deserts of North America: precipitation pulses, soil water, and plant responses. *Oecologia* 141 194–210. 10.1007/s00442-004-1524-4 15042457

[B66] Robertson T. R. Bell C. W. Zak J. C. Tissue D. T. (2009). Precipitation timing and magnitude differentially affect aboveground annual net primary productivity in three perennial species in a Chihuahuan Desert grassland. *New Phytol.* 181 230–242. 10.1111/j.1469-8137.2008.02643.x 19076724

[B67] Ryan M. G. Phillips N. Bond B. J. (2006). The hydraulic limitation hypothesis revisited. *Plant Cell Environ.* 29 367–381. 10.1111/j.1365-3040.2005.01478.x 17080592

[B68] Ryszkowski L. Kedziora A. (2007). Modification of water flows and nitrogen fluxes by shelterbelts. *Ecol. Engin.* 29 388–400. 10.1016/j.ecoleng.2006.09.023

[B69] Schulze E. D. Kelliher F. M. Korner C. Lloyd J. Leuning R. (1994). Relationships among maximum stomatal conductance, ecosystem surface conductance, carbon assimilation rate, and plant nitrogen nutrition - a global ecology scaling exercise. *Ann. Rev. Ecol. System.* 25 629–662. 10.1146/annurev.es.25.110194.003213

[B70] Schwinning S. Sala O. E. (2004). Hierarchy of responses to resource pulses in arid and semi-arid ecosystems. *Oecologia* 141 211–220. 15034778 10.1007/s00442-004-1520-8

[B71] Shen Q. Gao G. Y. Fu B. J. Lu Y. H. (2015). Sap flow and water use sources of shelter-belt trees in an arid inland river basin of Northwest China. *Ecohydrology* 8 1446–1458. 10.1002/eco.1593

[B72] Steppe K. Vandegehuchte M. W. Tognetti R. Mencuccini M. (2015). Sap flow as a key trait in the understanding of plant hydraulic functioning. *Tree Physiol.* 35 341–345. 10.1093/treephys/tpv033 25926534

[B73] Tie Q. Hu H. C. Tian F. Q. Guan H. D. Lin H. (2017). Environmental and physiological controls on sap flow in a subhumid mountainous catchment in North China. *Agric. Forest Meteorol.* 240 46–57. 10.1016/j.agrformet.2017.03.018

[B74] Wang D. Gao G. Y. Li J. R. Yuan C. Lu Y. H. Fu B. J. (2020). Sap flow dynamics of xerophytic shrubs differ significantly among rainfall categories in the Loess Plateau of China. *J. Hydrol.* 585:124815. 10.1016/j.jhydrol.2020.124815

[B75] Wang F. Pan X. Gerlein-Safdi C. Cao X. Wang S. Gu L. (2020). Vegetation restoration in Northern China: a contrasted picture. *Land Degradation Dev.* 31 669–676. 10.1002/ldr.3314

[B76] Wang G. Zhao W. Liu H. Zhang G. Li F. (2015). Changes in soil and vegetation with stabilization of dunes in a desert–oasis ecotone. *Ecol. Res.* 30 639–650. 10.1007/s11284-015-1267-1

[B77] Wang G. H. Shen Y. Y. Yang X. L. Chen Z. X. Mo B. R. (2019). Scaling Up Sap Flow Measurements from the Stem Scale to the Individual Scale for Multibranched Caragana Korshinskii on the Chinese Loess Plateau. *Forests* 10:785. 10.3390/f10090785

[B78] Wang P. Niu G. Y. Fang Y. H. Wu R. J. Yu J. J. Yuan G. F. (2018). Implementing Dynamic Root Optimization in Noah-MP for Simulating Phreatophytic Root Water Uptake. *Water Res. Res.* 54 1560–1575. 10.1002/2017wr021061

[B79] Wang P. Pozdniakov S. P. (2014). A statistical approach to estimating evapotranspiration from diurnal groundwater level fluctuations. *Water Res. Res.* 50 2276–2292. 10.1002/2013wr014251

[B80] Wang T. Song X. Yan C. Li S. Xie J. (2011). Remote Sensing Analysis on Aeolian Desertification Trends in Northern China during 1975—2010. *J. Desert Res.* 31 1351–1356.

[B81] Wang X. P. Schaffer B. E. Yang Z. Rodriguez-Iturbe I. (2017). Probabilistic model predicts dynamics of vegetation biomass in a desert ecosystem in NW China. *Proc. Natl. Acad. Sci. U.S.A.* 114 E4944–E4950. 10.1073/pnas.1703684114 28584097 PMC5488948

[B82] Wang Y. Shao M. A. Liu Z. (2010). Large-scale spatial variability of dried soil layers and related factors across the entire Loess Plateau of China. *Geoderma* 159 99–108. 10.1016/j.geoderma.2010.07.001

[B83] Weltzin J. F. Tissue D. T. (2003). Resource pulses in arid environments - patterns of rain, patterns of life. *New Phytol.* 157 171–173. 10.1046/j.1469-8137.2003.00672.x 33873639

[B84] Williams D. G. Cable W. Hultine K. Hoedjes J. C. B. Yepez E. A. Simonneaux V. (2004). Evapotranspiration components determined by stable isotope, sap flow and eddy covariance techniques. *Agric. Forest Meteorol.* 125 241–258. 10.1016/j.agrformet.2004.04.008

[B85] Xia J. Lang Y. Zhao Q. Liu P. Su L. (2021). Photosynthetic characteristics of Tamarix chinensis under different groundwater depths in freshwater habitats. *Sci. Total Environ.* 761:143221. 10.1016/j.scitotenv.2020.143221 33218805

[B86] Xu H. Li Y. (2006). Water-use strategy of three central Asian desert shrubs and their responses to rain pulse events. *Plant Soil* 285 5–17. 10.1007/s11104-005-5108-9

[B87] Xu H. Zhang X. Yan H. Liang S. Shan L. (2009). Plants water status of the shelterbelt along the Tarim Desert Highway. *Sci. Bull.* 53 146–155. 10.1007/s11434-008-6017-0

[B88] Xu X. Sun B. Ding G. Guo S. Chai C. (2008). Sap flow patterms of three main sand-fixing shurbs and their responses to environmental factors in desert areas. *Acta Ecol. Sin.* 03 895–905.

[B89] Yu T. Feng Q. Si J. Xi H. Li Z. Chen A. (2013). Hydraulic redistribution of soil water by roots of two desert riparian phreatophytes in northwest China’s extremely arid region. *Plant Soil* 372 297–308. 10.1007/s11104-013-1727-8

[B90] Yuan G. Zhang P. Shao M. A. Luo Y. Zhu X. (2014). Energy and water exchanges over a riparian Tamarix spp. stand in the lower Tarim River basin under a hyper-arid climate. *Agric. Forest Meteorol.* 194 144–154. 10.1016/j.agrformet.2014.04.004

[B91] Yue G. Zhang T. Liu X. Yi X. (2006). Development and Application of Thernal Method in Measuring Stem Sap Flow. *Sci. Silv. Sin.* 08 102–108.

[B92] Zeppel M. J. B. Murray B. R. Barton C. Eamus D. (2004). Seasonal responses of xylem sap velocity to VPD and solar radiation during drought in a stand of native trees in temperate Australia. *Funct. Plant Biol.* 31 461–470. 10.1071/fp03220 32688918

[B93] Zha T. S. Qian D. Jia X. Bai Y. J. Tian Y. Bourque C. P. A. (2017). Soil moisture control of sap-flow response to biophysical factors in a desert-shrub species. *Artem. Ordosica. Biogeosci.* 14 4533–4544. 10.5194/bg-14-4533-2017

[B94] Zhang Q. Y. Jia X. X. Shao M. G. Zhang C. C. Li X. D. Ma C. K. (2018). Sap flow of black locust in response to short-term drought in southern Loess Plateau of China. *Sci. Rep.* 8:6222. 10.1038/s41598-018-24669-5 29670221 PMC5906651

[B95] Zhang Y. Yang Z. Guo S. Wang Q. Zhan K. Zhang J. (2018). Ecological changes in the Minqin oasis belt over the past 20 years. *Acta Pratacult. Sin.* 27 14–24.

[B96] Zhao W. Z. Liu B. (2010). The response of sap flow in shrubs to rainfall pulses in the desert region of China. *Agric. Forest Meteorol.* 150 1297–1306. 10.1016/j.agrformet.2010.05.012

[B97] Zhou H. Zhao W. Yang Q. (2016). Root biomass distribution of planted Haloxylon ammodendron in a duplex soil in an oasis: desert boundary area. *Ecol. Res.* 31 673–681. 10.1007/s11284-016-1376-5

